# Facile preparation of air-stable n-type thermoelectric single-wall carbon nanotube films with anionic surfactants

**DOI:** 10.1038/s41598-020-64959-5

**Published:** 2020-05-15

**Authors:** Yuhei Seki, Kizashi Nagata, Masayuki Takashiri

**Affiliations:** Department of Materials Science, Tokai University, Hiratsuka, Kanagawa 259-1292 Japan

**Keywords:** Nanowires, Thermoelectric devices and materials

## Abstract

Thermoelectric generators based on single-wall carbon nanotubes (SWCNTs) have great potential for use in wearable and skin electronics because of their lightweight and mechanically soft structure. However, the fabrication of air-stable n-type thermoelectric SWCNTs using conventional processes is challenging. Herein, we propose a facile process for fabricating air-stable n-type SWCNT films with anionic surfactants via drop casting followed by heat treatment. We examined different surfactants (Sodium Dodecyl Sulfate, Sodium Dodecylbenzene Sulfonate, and Sodium Cholate) and heat-treatment temperatures. The optimal SWCNT film maintained the n-type Seebeck coefficient for 35 days. Moreover, to further extend the n-type Seebeck coefficient maintenance, we periodically reheated the SWCNT film with a surfactant that had returned to the p-type Seebeck coefficient. The reheated film recovered the n-type Seebeck coefficient, and the effect of the reheating treatment lasted for several reheating cycles. Finally, we elucidated a simple mechanism for realizing an air-stable n-type Seebeck coefficient based on spectroscopic analyses of the SWCNT films.

## Introduction

Thermoelectric generators, which produce electricity based on small temperature differences from the room temperature, have attracted considerable interest^1–3^ owing to the requirement for huge number of sensors in internet of things (IoT) applications^4,5^. Thermoelectric generators are promising sources of electric power for these sensors. They consist of p- and n-type semiconducting pillars connected by metal electrodes^6–8^. Electric power is generated by introducing a temperature difference between the hot and cold sides of the semiconducting pillars.

Thermoelectric generators used as power sources for sensors should exhibit high thermoelectric performance at temperatures close to the room temperature along with small size, flexibility, and low cost of materials. To satisfy these requirements, film-type thermoelectric generators using inorganic materials such as bismuth telluride based alloys have been investigated^9–14^. Certainly, inorganic materials exhibit high thermoelectric performance, but flexibility and material cost are causes for concern^15–17^. Therefore, conductive polymers and carbon nanotubes (CNTs) have been developed as thermoelectric materials^18–21^. These materials have high flexibility, and the material cost is lower than that of inorganic thermoelectric materials. In particular, single-walled carbon nanotube (SWCNT) is a very promising material for thermoelectric devices, and other fields such as fuel cells, solar cells, and so on^22–27^. SWCNTs represent a unique 1D carbon allotrope with structural, electrical, and thermal properties that enable efficient thermoelectric energy conversion using the quantum-confinement effect^28–31^.

Although SWCNTs demonstrate great potential as thermoelectric materials, they exhibit p-type semiconducting property when exposed to air because of oxygen atoms that combine with the surface of the SWCNTs^32^. Therefore, the development of air-stable n-type SWCNTs has been challenging for many researchers^33–35^. Lee *et al*. reported early promising cases where alkali-metal doping contributed to change in the n-type property, but it was difficult to maintain the property under ambient conditions^36^. Later, SWCNTs were converted into air-stable n-type property using noncovalent functionalization with organic nitrogen-containing electron donors^37–39^. Nonoguchi *et al*. reported that salt-coordinated n-type SWCNTs displayed remarkable air stability for an extended period, even at 100 °C^40^. Zhou *et al*. recently reported that pristine p-type SWCNT films could be rapidly and conveniently switched to air-stable n-type films by drop casting a liquid dispersion of polyethyleneimine in ethanol^41^. These pioneering studies encouraged us to explore a facile method for obtaining n-type SWCNTs with long-term stability as well as identify the underlying mechanism for the n-type thermoelectric property.

Consequently, we propose a facile preparation method for n-type SWCNT films using surfactants. We prepared different types of anionic surfactants, which were added to a liquid dispersion of SWCNTs and used as dopants for charge carrier alternation. The SWCNT films were fabricated by drop-casting method using the liquid containing the SWCNTs and the surfactant, followed by heat treatment. To clarify the mechanism for exhibiting the n-type property, we analyzed various aspects of the structures of the SWCNT films. The stability of the thermoelectric properties under atmospheric conditions was evaluated near 300 K for approximately two months. In addition, we periodically reheated the SWCNT films after two months to investigate whether they recovered the n-type property.

## Results

### Structural properties of SWCNT films with surfactants

**Figure 1:**
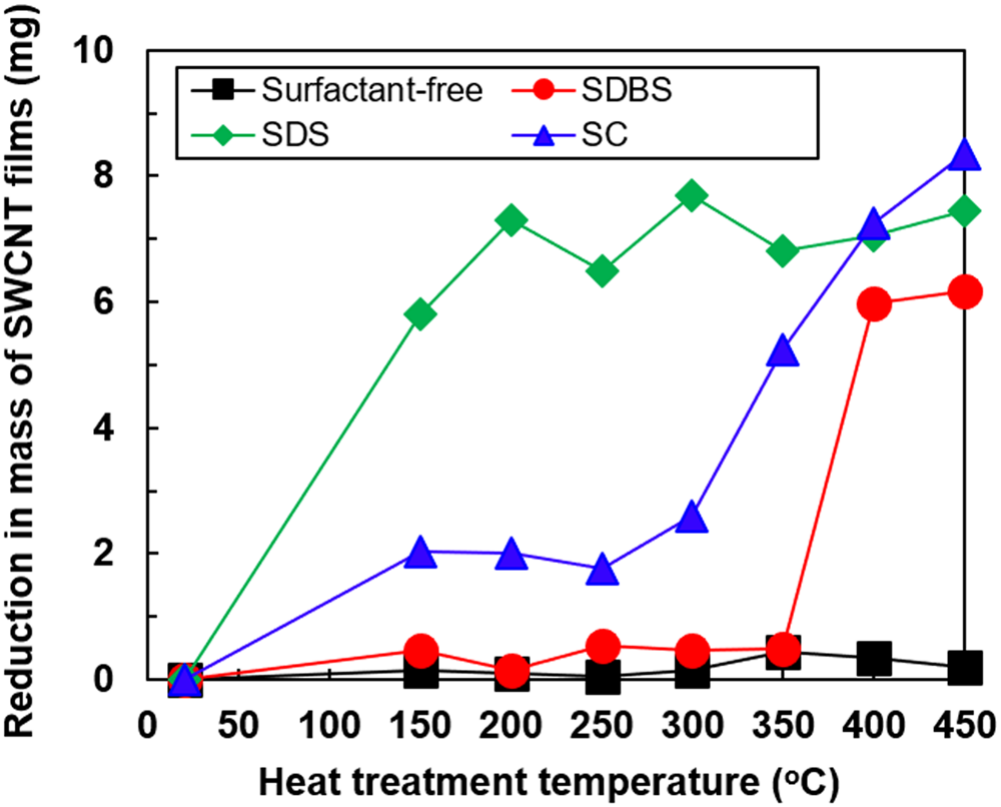
Reduction in mass of SWCNT films with different surfactants as a function of heat-treatment temperature.

**Figure 2:**
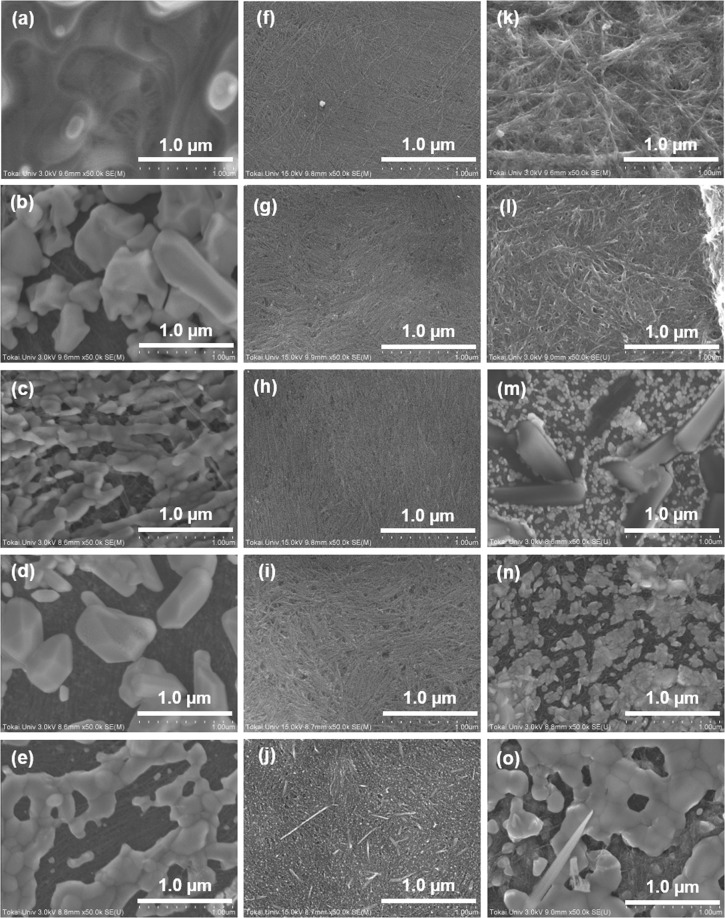
Surface morphologies of SWCNT films with different surfactants and heat-treatment temperatures, observed with SEM. (**a**) SDS-non heated. (**b**) SDS-150 °C. (**c**) SDS-250 °C. (**d**) SDS-350 °C. (**e**) SDS-450 °C. (**f**) SDBS-non heated. (**g**) SDBS-150 °C. (**h**) SDBS-250 °C. (**i**) SDBS-350 °C. (**j**) SDBS-450 °C. (**k**) SC-non heated. (**l**) SC-150 °C. (**m**) SC-250 °C (**n**) SC-350 °C. (**o**) SC-450 °C.

We used three types of anionic surfactants, namely, sodium dodecyl sulfate (SDS), sodium dodecylbenzenesulfonate (SDBS), and sodium cholate (SC). Their molecular structures are shown in Supplementary Fig. 1. To investigate the evaporation of the surfactant from the SWCNT films, we measured the mass of the samples with and without heat-treatment and then calculated the difference in mass between the samples, as shown in Fig. 1. The mass of the surfactant-free SWCNT films were not changed at any of the heat-treatment temperatures, indicating that the heat treatment using the mixture of argon (95%) and hydrogen (5%) gases prevented the surface oxidation of the SWCNTs. In the SWCNT films with SDS surfactant, the reduction in mass began at 150 °C, and it was saturated at over 200 °C. The reduction in mass of the SWCNT films with SC surfactant was observed at 300 °C, and the magnitude of the reduction at 400 °C was almost the same between the SWCNTs with SC and SDS. On the other hand, the reduction in mass of the SWCNT films with SDBS surfactant was not observed until the heat-treatment temperature of 350 °C, and the mass reduction became prominent over the temperature of 400 °C. Therefore, SDBS has the highest ability among the three surfactants to retain the surfactant elements in the SWCNT films. A surface SEM image of the surfactant-free SWCNT film is presented in Supplementary Fig. 2, and the surface SEM images of SWCNT films with different surfactants and subjected to different heat-treatment temperatures are shown in Fig. 2. The surface of the SWCNT film with no heat-treatment was mostly covered with the solid material of the SDS surfactant (Fig. 2a). When the heat-treatment temperature was increased to 150 °C, random-shaped crystal-like structures were formed on the surface and the underlying SWCNT surface could be partially observed (Fig. 2b). This indicated that SDS was not homogeneously mixed with the SWCNT. Even when the heating temperature was further increased, the essential surface morphology was not much different from that at 150 °C, but the area covered by the crystal-like structures seemed to gradually decrease (Fig. 2c–e), which corresponded to the reduction in mass of the SWCNT films with SDS surfactant, as shown in Fig. 1.

When using the SDBS surfactant, SDBS and SWCNTs were homogeneously mixed and the surface was relatively flat in the film without heat treatment (Fig. 2f). The surface morphology was not changed significantly until the heat-treatment temperature of 350 °C (Fig. 2g–i). At the heat-treatment temperature of 450 °C, SDBS was evaporated from the surface (Fig. 2j). This phenomenon was also supported by the reduction in mass of the SWCNT films with SDBS surfactant, as shown in Fig. 1.

When SC was used as the surfactant, the solid material of the surfactant clung on to the SWCNTs in the film with no heat-treatment (Fig. 2k), and the surface roughness of the film became larger than that of the film with SDBS surfactant. At the heat-treatment temperature of 150 °C, the surface roughness became small owing to evaporation of excessive surfactant (Fig. 2l). When the heat-treatment temperature was increased to 250 °C, the surface morphology drastically changed. Small and large crystal-like structures appeared on the film surface (Fig. 2m). Upon further increasing the heat-treatment temperature, the small crystal-like structures coalesced and enlarged (Fig. 2n–o).

Therefore, we found that the surface morphology of the films greatly depended on the type of surfactant. SDBS was well mixed with the SWCNTs and evaporated from the film surface with great difficulty. In contrast, SDS separated from the SWCNTs and easily evaporated from the film surface. The cross-section SEM images of the SWCNT films, provided in Supplementary Fig. 3, clearly show the well-mixing of the SDBS surfactant with the SWCNTs and the separating layers of the SDS and SC surfactants. This difference was possibly because the SDBS solution had the highest wettability among the three types of surfactants, which contributed to the penetration of the surfactant into the gaps of the SWCNTs. The results of the wettability measurements of the surfactant solutions are illustrated in Supplementary Fig. 4.

### Seebeck coefficient of SWCNT films with surfactant

**Figure 3:**
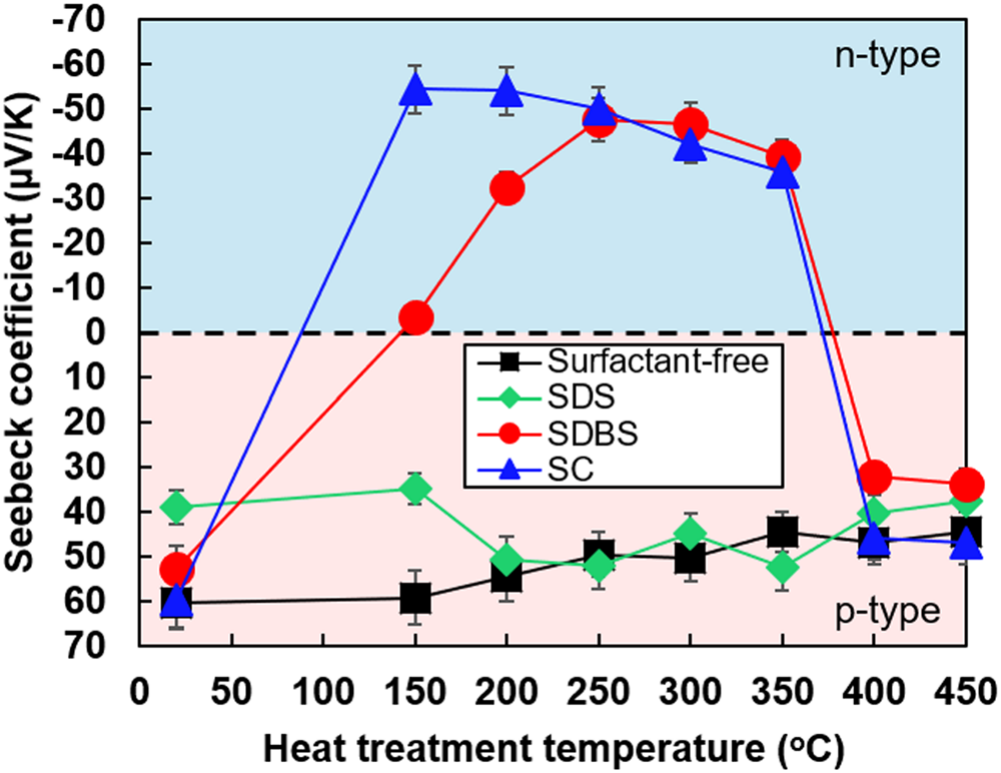
Seebeck coefficient of SWCNT films with different surfactants as a function of heat-treatment temperature.

Figure 3 shows the Seebeck coefficient of SWCNT films with different surfactants as a function of the heat-treatment temperature. It is to be noted that the Seebeck coefficients of SWCNT films receiving no heat treatment were plotted for a temperature of 20 °C for convenience. The Seebeck coefficient of the surfactant-free SWCNT film was 60 μV/K with no heat treatment. Even though the Seebeck coefficient slightly decreased with increase in the heat-treatment temperature, the SWCNT films maintained a relatively large p-type Seebeck coefficient at all heat-treatment temperatures.

When SDS was used as the surfactant, the Seebeck coefficient of the SWCNT film did not exhibit n-type property at any of the heat-treatment temperatures. The trend of the SWCNT films with SDS was similar to that of the surfactant-free SWCNT film.

On the other hand, the Seebeck coefficient of the SWCNT films with SDBS negatively increased with increase in the heat-treatment temperature. It became a negative value at the heat-treatment temperature of 150 °C; thereafter, the negative value increased and saturated at approximately −50 μV/K at heat-treatment temperatures between 250 °C and 350 °C. With further increase in the heat-treatment temperature, the Seebeck coefficient became positive again.

The SWCNT films with SC converted to n-type Seebeck coefficient at a lower heat-treatment temperature than that observed for the SWCNT film with SDBS. The negative maximum value of the Seebeck coefficient was −54 μV/K at the heat-treatment temperature of 150 °C. The Seebeck coefficient slightly decreased negatively with increase in the heat-treatment temperature. The trend of the Seebeck coefficient was almost same as that of the film with SDBS at heat-treatment temperatures between 250 °C and 350 °C. At a higher heat-treatment temperature, the Seebeck coefficient of the film with SC converted to positive values again.

Therefore, the SDBS and SC surfactants contributed to converting the Seebeck coefficient to n-type property at medium heat-treatment temperatures, but excessive heat treatment restored the p-type property of the Seebeck coefficient. The relationship between resistance of the SWCNT films with different surfactants and the heat-treatment temperature is depicted in Supplementary Fig. 5. We have displayed the resistance instead of the electric conductivity because it is difficult to evaluate the effective film thickness of SWCNT films with surfactants. Here, the effective film thickness is the value assuming that the porosity of the film is 0% whereas the visual film thickness determined by the cross-section SEM image includes pores and gaps in the SWCNT films.

### Stability of n-type Seebeck coefficient in SWCNT films with surfactants

**Figure 4:**
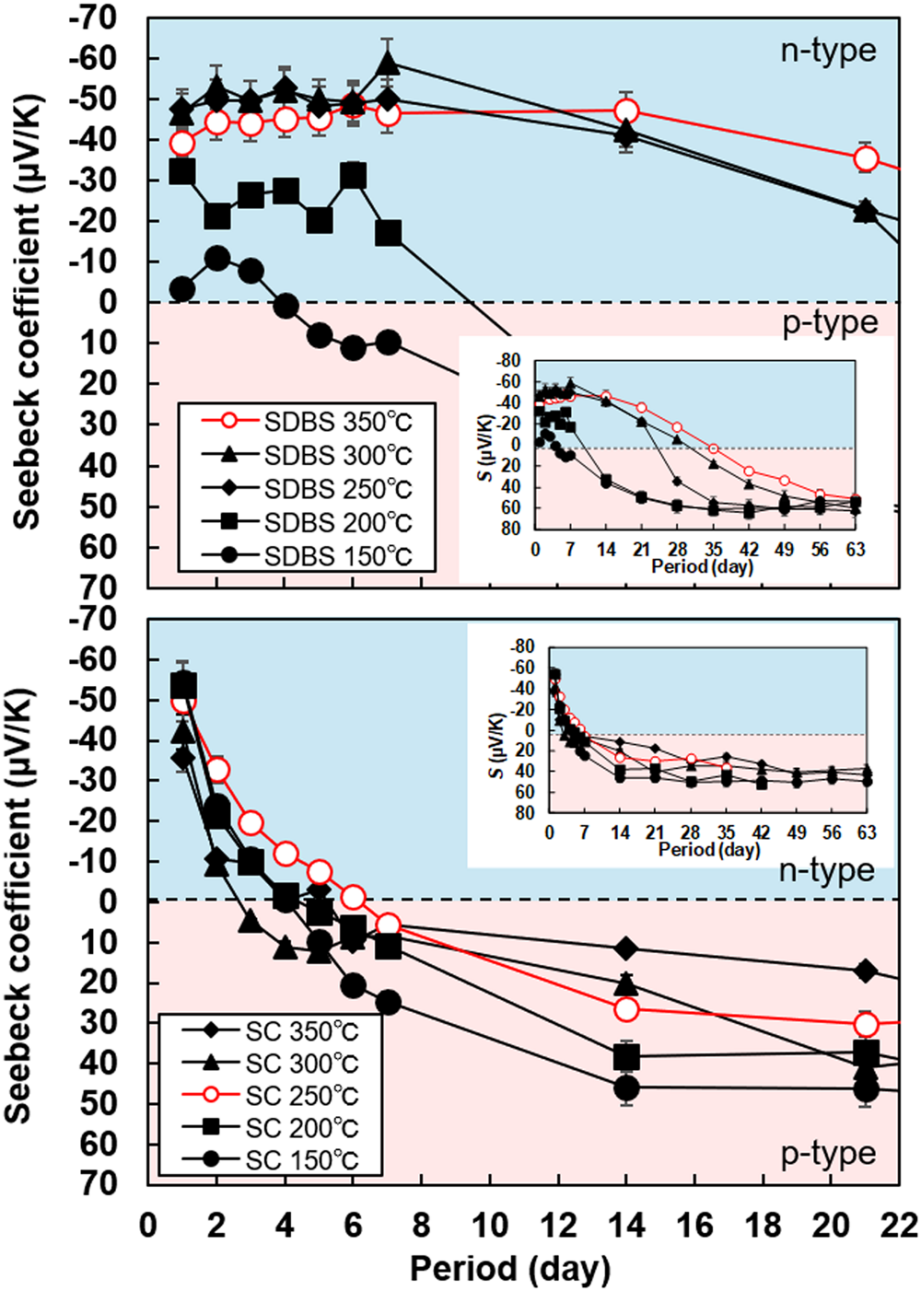
Chronological change in Seebeck coefficients of SWCNT films with different surfactants and heat-treatment temperatures. (**a**) SWCNT films with SDBS. (**b**) SWCNT films with SC.

Figure 4 shows the change in the Seebeck coefficient of SWCNT films with different surfactants over a period of 21 days. In addition, we measured the chronological change in the Seebeck coefficient for up to 63 days, and the results are depicted in the insets. Here, the Seebeck coefficients of only the SWCNT films with SDBS and SC were measured because the films with SDS did not exhibit n-type Seebeck coefficient. In addition, the change in the Seebeck coefficients of the SWCNT films heated above 400 °C were not measured because the Seebeck coefficient showed p-type property again.

In Fig. 4a, the SWCNT films with SDBS exhibited negative value of the Seebeck coefficient, and the duration of the n-type property increased as the heat-treatment temperature was increased from 150 °C to 250 °C. When the heat-treatment temperature was in the range of 250 °C to 350 °C, the Seebeck coefficient was approximately −50 μV/K for 14 days. Thereafter, it gradually degraded and finally showed p-type property. The SWCNT films heated at 350 °C exhibited the highest stability, where the n-type Seebeck coefficient was maintained for 35 days. The Seebeck coefficients of all the samples after 63 days became approximately 60 μV/K (p-type), which was the same value as that of surfactant-free SWCNT films. Figure 4b shows that the Seebeck coefficient of SWCNT films with SC rapidly degraded with time. The highest stability was observed in the film heated at 250 °C but the Seebeck coefficient exhibited p-type property after only 6 days. These results showed that the stability of the n-type Seebeck coefficient strongly depended on the type of surfactant and the optimum heat-treatment temperature was in the range of 250 °C to 350 °C. Thus, we fabricated SWCNT films that exhibited relatively high stability of n-type Seebeck coefficient by using a facile process with an appropriate surfactant. The chronological change in resistance of the SWCNT films with different surfactants is provided in Supplementary Fig. 6. We also estimated the chronological change in power factor of SWCNT films with SDBS as surfactant and heat treatment at 350 °C, which was the best condition in this study, as provided in Supplementary Fig. 7. The power factor was estimated based on the visual film thickness determined by the cross-section SEM images. The highest power factor of 7.1 μW/(m⋅K^2^) was observed on the first day. The power factor decreased until day 35 owing to the increase in the resistance of the film. Thereafter, the power factor increased while the resistance decreased, and the power factor was 3.9 μW/(m⋅K^2^) on day 63.

**Figure 5:**
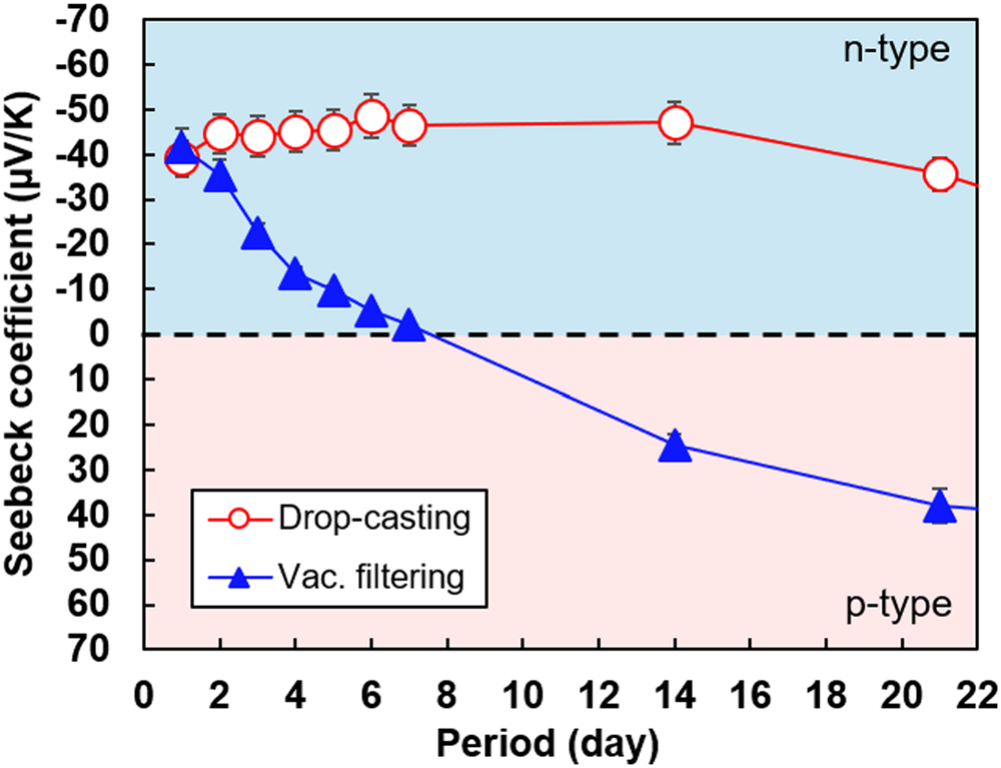
Chronological change in Seebeck coefficients of SWCNT films prepared by different fabrication methods with SDBS as the surfactant and heat treatment at 350 °C.

We believe that the drop casting process contributed to the stability of the n-type Seebeck coefficient in the SWCNT films. To demonstrate it, we prepared an SWCNT film with SDBS using the vacuum filtration process, which is a typical method for fabricating CNT films^42–44^, and compared the stability of the Seebeck coefficients in the two film-preparation methods. Figure 5 shows the chronological change in the Seebeck coefficient of SWCNT films prepared using drop casting and vacuum filtration. We employed SDBS as the surfactant and the heat-treatment temperature was 350 °C. The SWCNT film fabricated using the drop casting method exhibited the highest stability of the n-type Seebeck coefficient. The initial Seebeck coefficients of the SWCNT films fabricated using both methods were approximately the same. However, the Seebeck coefficient of the SWCNT film fabricated by vacuum filtering gradually degraded with time. It showed p-type property at 7 days, which was one-fifth of the corresponding time observed for the film fabricated using drop casting. This was attributed to the difference in the method employed to remove the surfactant during the film formation. A schematic diagram of the dispersion liquid removal technique in each film formation method is presented in Supplementary Fig. 8. The drop-casting method mainly evaporates the water while the surfactant remains in the film; thus, the resulting SWCNT film is rich in surfactants. Conversely, in the vacuum filtration method, the surfactant is sucked to remove water from the film, i.e., the surfactant is also removed along with the water. As a result, the resulting SWCNT film has poor surfactant content. This study did not investigate the amount of surfactant required for n-type doping, but the surfactant content contributed to the stability of the n-type Seebeck coefficient^45^.

**Figure 6:**
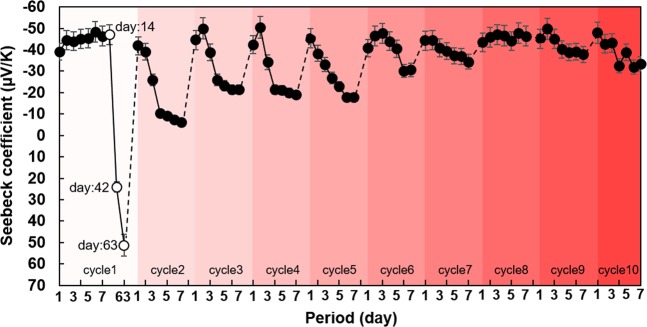
Recovery of Seebeck coefficients of SWCNT films with SDBS as surfactant and heat treatment at 350 °C. Cycle 1 corresponds to the result of the SWCNT film with SDBS heated at 350 °C, as shown in Fig. 3.

The SWCNT films were reheated to further investigate the stability of the n-type Seebeck coefficient in films prepared by drop casting. We used heat-treated SWCNT films with SDBS, which had exhibited the highest stability of the n-type Seebeck coefficient but degraded to the p-type Seebeck coefficient after 63 days. Figure 6 shows the transition of the Seebeck coefficient of the SWCNT films due to reheating. The reheat-treatment temperature was 350 °C for 1 h in Ar (95%)-H_2_ (5%) mixture gas, and the interval of the reheat-treatment was 7 days. Cycle 1 corresponds to the result of the SWCNT film with SDBS heated at 350 °C, as shown in Fig. 3. It was found that the n-type Seebeck coefficient of −42 μV/K was restored in the SWCNT film after the reheat-treatment in cycle 2. However, the Seebeck coefficient rapidly dropped and approached zero during the 7-day period. When the reheat-treatment was performed again (cycle 3), the film recovered the Seebeck coefficient of −44 μV/K. After 7 days, the Seebeck coefficient of the film was −22 μV/K, which was higher than that of the film at 7 days in cycle 1. Thereafter, when reheating was repeated, the Seebeck coefficient at the time of recovery (1 day) was a similar value, but it gradually increased after 7 days. This indicated that the stability of the n-type Seebeck coefficient was improved by repeating the heat treatment. The resistance of the SWCNT films decreased with time, as shown in Supplementary Fig. 9.

To clarify the underlying mechanism for recovering the n-type Seebeck coefficient by reheating, we measured the change in mass of the SWCNT films with time. The results are shown in Supplementary Fig. 10. Overall, the mass increased with time when the film was exposed to air, and it decreased after the re-heat treatment. Detailed analysis showed that the rate of increase of mass decreased when the number of re-heat treatments was increased. This indicated that the amount of oxygen adsorption was reduced with the number of heat treatments. Therefore, we concluded that this phenomenon occurred because the surfactant was tightly linked with the SWCNTs, which prevented surface oxidation^45,46^.

### Spectroscopic analyses of SWCNT films with surfactants

**Figure 7:**
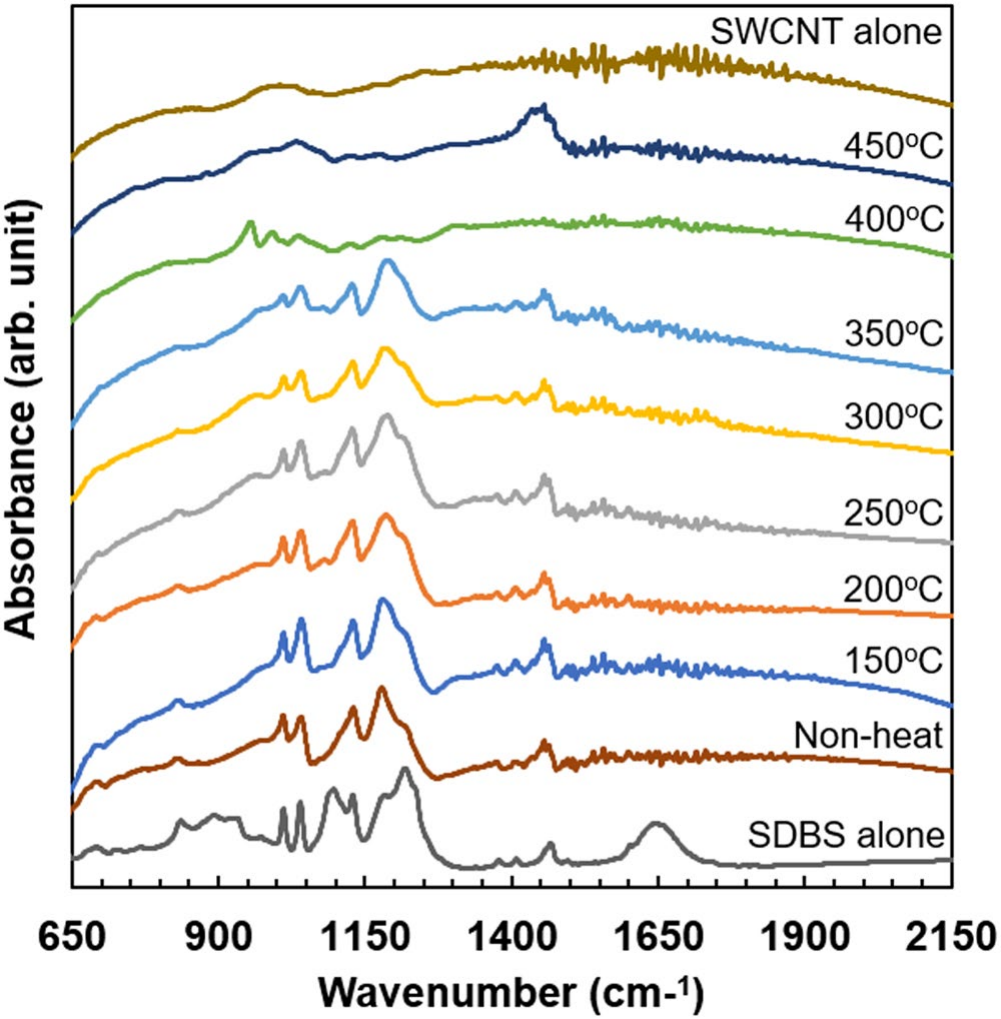
FT-IR spectra of SWCNT films with SDBS and different heat-treatment temperatures.

To clarify the mechanism by which the n-type Seebeck coefficient of SWCNT films remained stable by adding the optimal surfactant (SDBS), we analyzed the interaction between the SWCNTs and surfactant using spectroscopic analyses. First, FT-IR analysis was used to investigate the doping level of the surfactant in the SWCNT films. Figure 7 shows the IR spectra of the SWCNT films with SDBS surfactant, subjected to different heat-treatment temperatures. For comparison, the IR spectra of the SDBS and the surfactant-free SWCNT film are added in the figure. The IR spectra of the samples with no heat treatment and those heated from 150 °C to 350 °C were similar, which demonstrated two peaks corresponding to SDBS and SWCNT. This trend indicated that the SWCNTs and SDBS were homogeneously combined in the films. When the heat-treatment temperature was increased to over 400 °C, the absorbance peaks derived from SDBS decreased and the peaks derived from SWCNTs became dominant. This was because the elements of SDBS were gradually evaporated from the film surface; this phenomenon corresponds to the reduction in mass of the samples in Fig. 1. From the Seebeck coefficient measurements and FT-IR analysis with regard to the heat-treatment temperature, we conclude that the n-type Seebeck coefficient appeared when the SDBS elements remained in the SWCNT films. However, a mechanism for stabilizing the n-type Seebeck coefficient and identification of the responsible elements in SDBS are not yet clear from the FT-IR analysis.

**Figure 8:**
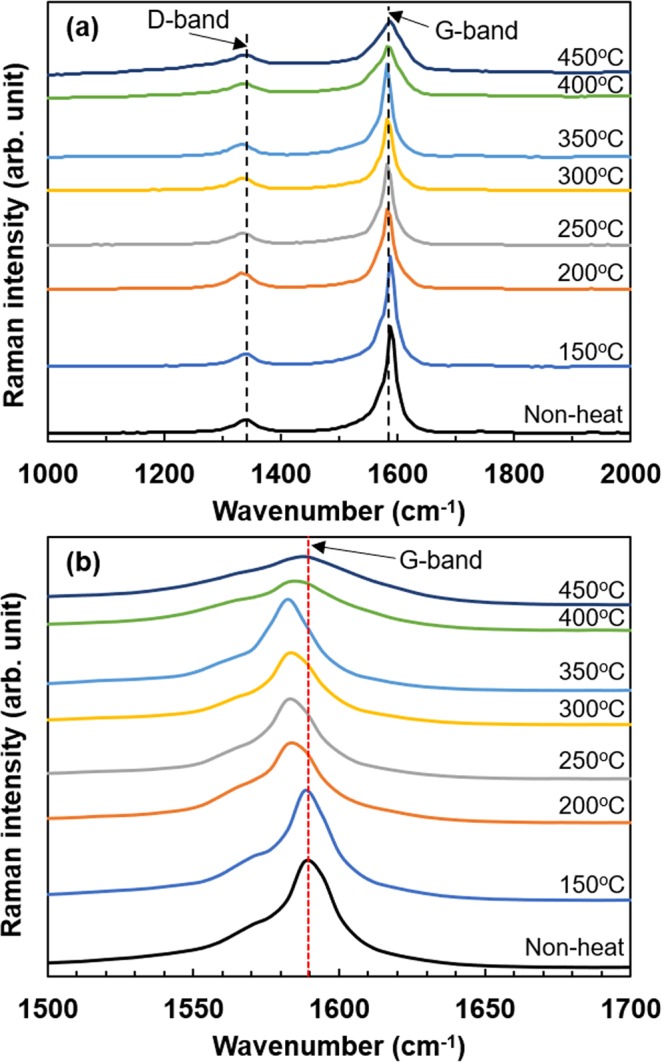
Raman spectra of SWCNT films with SDBS and different heat-treatment temperatures. (**a**) Wavenumber ranging from 1000 to 2000 cm^−1^. (**b**) Wavenumber ranging from 1500 to 1700 cm^−1^ for precisely displaying the G-band spectra.

Second, Raman spectroscopy was performed to confirm whether new bonds occurred between the SWCNT and SDBS to clarify the stabilization of the n-type Seebeck coefficient. Figure 8 shows the Raman spectra of SWCNT films with SDBS surfactant at different heat-treatment temperatures. In Fig. 8(a), clear peaks other than those of the defect-derived D-band and graphene-derived G-band were not observed at any of the temperatures. This indicates that no new bond occurred between the SDBS and SWCNT. However, when the scale of the wavenumber (*x*-axis) was enlarged, as shown in Fig. 8(b), the peak of the G-band shifted to a lower wavenumber as the heat-treatment temperature was increased to 350 °C. With further increase in the temperature, the peak of the G-band shifted to a higher wavenumber, and then the peak position of the G-band in the sample of 450 °C was equivalent to that of the non-heated sample. These peak shifts were not observed in the SWCNT films with SC surfactant at any of the heat-treatment temperatures, as shown in Supplementary Fig. 11. Thus, the peak shift of the G-band and the stabilization of n-type Seebeck coefficient have a strong correlation, i.e., a large peak shift increases the stability of the n-type Seebeck coefficient. The peak shift originally appears when the C–C bond expands (or contracts) and the electronic structure changes^47,48^. In this case, we believe that the SDBS components are tightly linked to the surface of the SWCNTs via an electrostatic force, resulting in the expansion of the C–C bond and change in the electronic structure.

**Figure 9:**
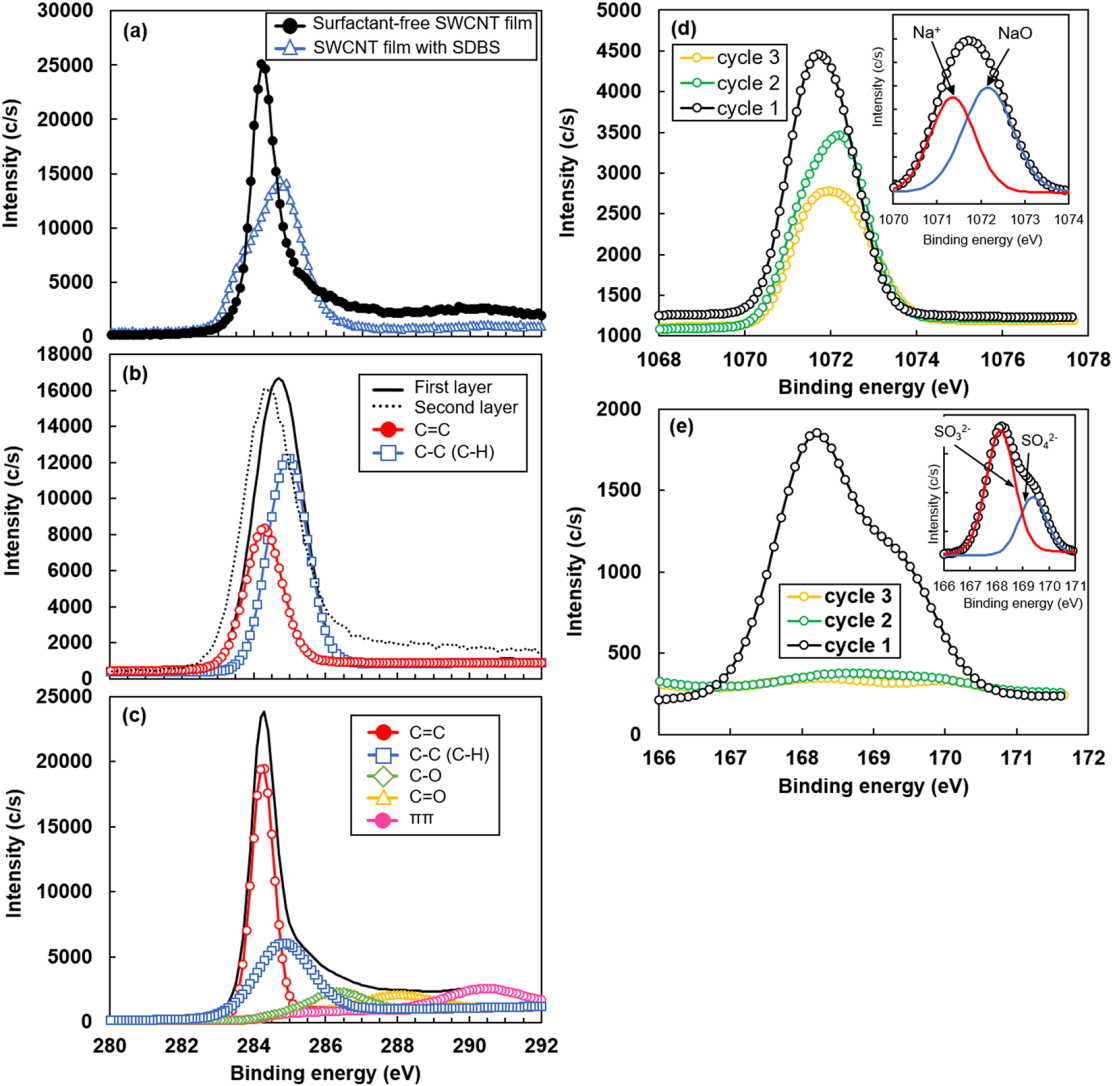
XPS spectra of SWCNT film with SDBS and surfactant-free SWCNT film with heat treatment at 350 °C. (**a**) C1s spectra of SWCNT film with SDBS and surfactant-free SWCNT film. (**b**) Decomposition and fitting of C1s spectra of SWCNT film with SDBS. (**c**) Decomposition and fitting of C1s spectra of surfactant-free SWCNT film. (**d**) Na1s spectra of SWCNT film with SDBS. (**e**) S2p spectra of SWCNT film with SDBS.

Third, XPS analysis was performed to investigate the surface condition of the SWCNT films with SDBS surfactant. Prior to the analysis of the SWCNT films with SDBS, we investigated the adsorption of oxygen on the SWCNT surface to compare the peak intensities of the O1s spectrum of the surfactant-free SWCNT film with no heat treatment and that with heat treatment at 350 °C. Because of the heat treatment, a certain amount of oxygen atoms desorbed from the SWCNT surface, as shown in Supplementary Fig. 12. Next, we analyzed the surface states of the SWCNT films using the C1s spectra of the surfactant-free SWCNT film and the SWCNT film with SDBS heated at 350 °C (Fig. 9a). The peak of the SWCNT film with SDBS showed a greater shift to the higher energy side than that of the surfactant-free SWCNT film. For detailed analysis, the C1s spectra were decomposed and fit to the Gaussian curves shown in Fig. 9b,c. In the SWCNT film with SDBS (Fig. 9b), the C—C (C—H) bond at 285 eV, derived from SDBS, was clearly detected, and its intensity was higher than that of the C=C bond derived from SWCNT. The depth analysis shown in this figure indicated that only the spectrum from the first layer (surface) was shifted to the higher energy side, and the peak position of the spectrum from the second layer corresponded to that from the surfactant-free SWCNT film, as shown in Fig. 9c. Therefore, the elements of SDBS were localized on the SWCNT surface. To reinforce the findings, the core spectral levels of Na1s and S2p were also analyzed by XPS. Figure 9d shows the core spectral level of Na1s. The intensity on the lower energy side mainly decreased as the number of cycles increased. As shown in the inset, the spectrum in cycle 1 was separated into two peaks, namely, Na^+^ and NaO. Accordingly, the peak intensity of Na^+^ mainly decreased as the number of cycles increased. Figure 9e shows the core spectral level of S2p. The core spectra of S2p showed a large peak in the region from 167 to 170 eV on the surface (cycle 1). This peak was separated into two SO_3_^2−^ and SO_4_^2−^peaks, as shown in the inset. The two peaks drastically decreased with increase in the number of cycles, indicating that sulfur oxide ions were mainly localized on the SWCNT surface. These results indicate that SDBS covered the surface of the SWCNT films.

## Discussion

**Figure 10:**
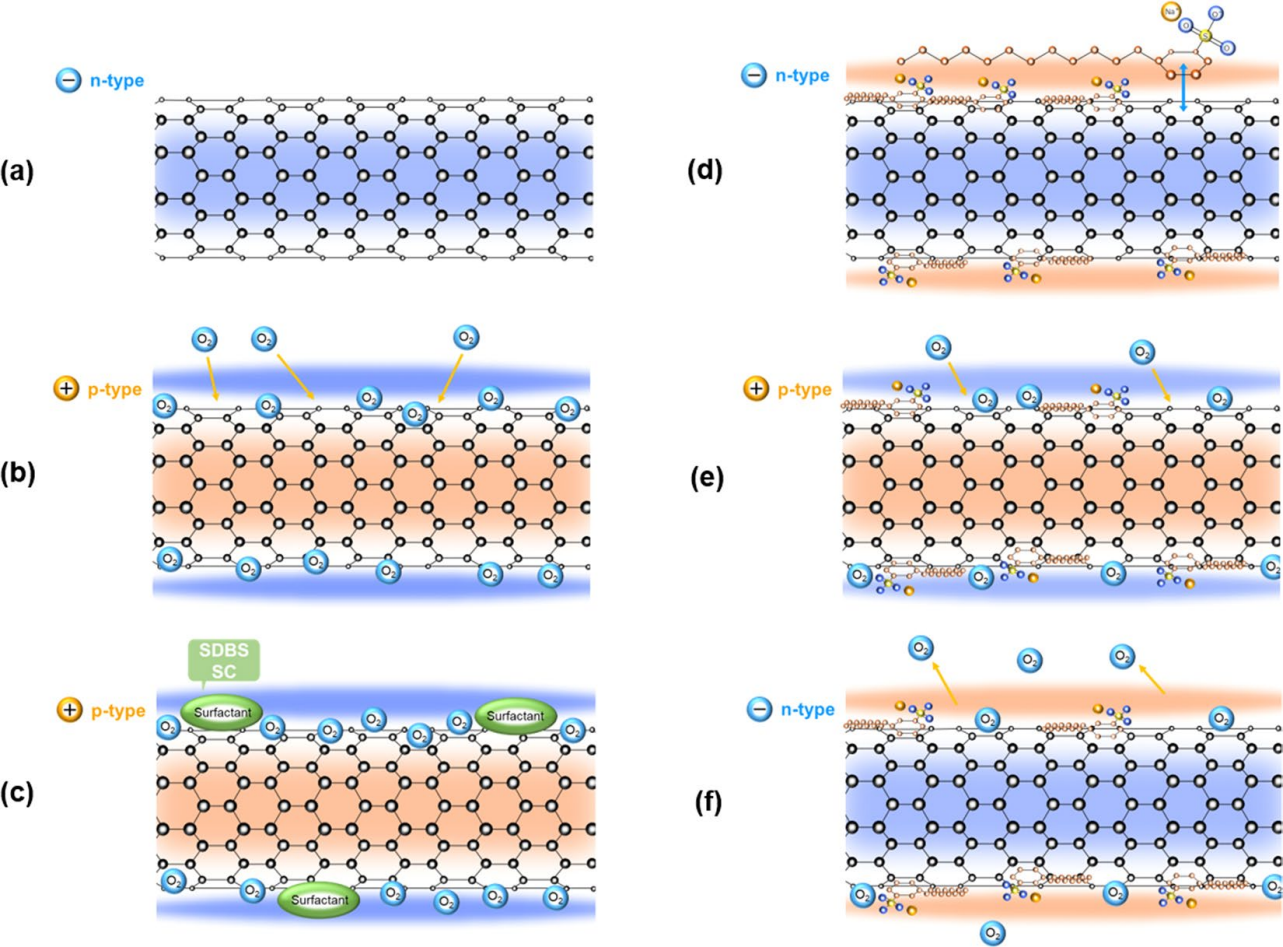
Schematic diagram of the mechanism of air-stable n-type Seebeck coefficient exhibited in SWCNT films. (**a**) Pristine SWCNTs. (**b**) Surfactant-free SWCNTs. (**c**) Non-heated SWCNTs homogeneously covered with SDBS surfactant. (**d**) Heated SWCNTs homogeneously covered with SDBS surfactant. (**e**) Heated SWCNTs homogeneously covered with SDBS surfactant over a period of time. (**f**) Reheated SWCNTs homogeneously covered with SDBS surfactant.

From the above-mentioned measurements and analyses, we consider a simple mechanism by which the SWCNT films with SDBS surfactant exhibits air-stable n-type Seebeck coefficient under heat treatment and the Seebeck coefficient recovers from its initial value upon reheating. Figure 10 shows a schematic diagram of the mechanism that supports air-stable n-type Seebeck coefficient of SWCNT films with SDBS surfactant. Initially, the pristine SWCNTs, which are not covered with oxygen atoms, exhibit n-type Seebeck coefficient^46^, as depicted in Fig. 10a. When the SWCNTs are exposed to air, oxygen atoms are adsorbed on the SWCNT surface, which corresponds to the state of surfactant-free SWCNT films (Fig. 10b). This phenomenon causes electrons to transfer from SWCNTs to oxygen atoms, thus decreasing the Fermi energy. As a result, the Seebeck coefficient shows p-type property. Figure 10c illustrates the condition where the SWCNTs are homogeneously covered with the SDBS surfactant and are not heated. Because the oxygen atoms are adsorbed on the SWCNT surface, the Seebeck coefficient exhibits p-type property. When the SWCNT films with SDBS are heated, oxygen atoms desorb from the SWCNT surface and the elements of SDBS get absorbed on the SWCNT surface, as shown in Fig. 10d. Unlike oxygen atoms, elements of SDBS cannot transfer the electrons from the SWCNT. This condition is equivalent to that of pristine SWCNTs; thus, the Seebeck coefficient exhibits n-type property. The exact reason that the SDBS surfactant is strongly linked to the SWCNT surface has not been clarified yet. However, we consider that sodium cations are attracted to the SWCNT surface by electrostatic force. In addition, the presence of sulfur oxide ions and benzene ring structure in SDBS might contribute to enhanced binding. Here, it is to be noted that the SDS surfactant cannot achieve n-type Seebeck coefficient, whereas the SC surfactant can achieve n-type Seebeck coefficient but its stability is low, as shown in Fig. 3. One of the reasons is that the SDS and SC surfactants are not sufficiently mixed with the SWCNTs; therefore, the elements of the surfactant are mostly located on the surface of the SWCNTs, as shown in Fig. 2. As shown in Fig. 10e, the Seebeck coefficient of the heated SWCNT film with SDBS changes from n-type to p-type property over a period of time. This is because oxygen atoms re-enter the SWCNT surface and combine with the carbon atoms. However, the reheating treatment eliminates the oxygen atoms, and the n-type Seebeck coefficient is restored by binding of the SDBS elements to the SWCNT again, as shown in Fig. 10f.

This work not only proposes a facile process for fabricating n-type SWCNT films, but also reveals extensive prospects for its real-time application. An all-carbon thermoelectric generator, which consists of multiple p–n couples, can be realized by fabricating n-type SWCNT films. Moreover, because the proposed method can control the p-type and n-type Seebeck coefficients only by the presence or absence of a surfactant, the two types of films can be fabricated via almost the same process. This can simplify the fabrication process of generators, thus reducing its manufacturing cost. Even though the retention time of the n-type Seebeck coefficient is currently limited, the property recovers with reheating. In the future, we plan to prolong the reheating interval and eventually develop a process that does not require reheating. One approach involves coating the thermally heated SWCNT films with surfactant by an insulating layer to prevent oxygen atoms from recombining with the surface of the SWCNTs. A layer having low thermal conductivity is desirable for the insulating layer because a temperature difference is easily created in the thermoelectric generator.

In summary, n-type SWCNT films with air-stable n-type Seebeck coefficient were achieved using a facile process. The anionic surfactants were mixed with SWCNTs, and the films were formed using drop casting followed by heat treatment. We examined the effectiveness of three types of anionic surfactants (SDS, SDBS, and SC). When SDBS was used as the surfactant and the heat-treatment temperature was set at 350 °C, the Seebeck coefficient showed n-type property, which was maintained for 35 days. One of the reasons for this phenomenon was that SDBS and the SWCNTs were homogeneously mixed in the dispersion liquid and the SDBS elements were tightly linked to the surface of the SWCNTs. Moreover, to further prolong the duration of the n-type Seebeck coefficient, we periodically reheated the SWCNT film with SDBS, which had already returned to the p-type Seebeck coefficient. The reheated film recovered the n-type Seebeck coefficient, and the effect of reheating continued for many reheating cycles.

## Methods

### Materials

Single-wall carbon nanotube (SWCNT), called super growth-carbon nanotube (ZEONANO SG101), was supplied by Zeon Corporation. The anionic surfactants, namely, sodium dodecyl sulfate (SDS; FUJIFILM Wako Pure Chemical), sodium dodecylbenzenesulfonate (SDBS; Tokyo Chemical Industry Co.), and sodium cholate (SC; FUJIFILM Wako Pure Chemical), were used as received.

### Preparation of SWCNT films with surfactants

The SWCNTs added with surfactants were ultrasonically dispersed in deionized water at a concentration of 0.2 wt%. We used three types of anionic surfactants, namely, SDS, SDBS, and SC. Their molecular structures are provided in Supplementary Fig. 1. For comparison, we also prepared surfactant-free SWCNT films, which were dispersed in ethanol at a concentration of 0.2 wt%. The SWCNT films were prepared on a glass substrate with a limited deposition area (2.5 cm × 2.0 cm) using the drop-casting method. A pipette was used to drop cast 0.9 ml of the SWCNT dispersion liquid on the substrate. After drop casting, the dispersion liquid was naturally dried under atmospheric conditions for approximately 24 h.

The SWCNT films deposited on the glass substrate were heated using an electric furnace. The detailed procedures for heat treatment have been presented in our previous reports^49,50^. In brief, the furnace was filled with a mixture of argon (95%) and hydrogen (5%) gases at atmospheric pressure. We added hydrogen gas to reduce the oxygen atoms on the SWCNT surface. The heat-treatment temperatures were set at 150, 200, 250, 300, 350, 400, and 450 °C and the treatment time was maintained for 1 h. The sample was taken out when the temperature in the furnace reached less than 100 °C. The difference in mass between the samples with and without heat-treatment was calculated, as shown in Fig. 1, to determine the amount of surfactant evaporated from the SWCNT films.

### Measurement of thermoelectric properties

The in-plane Seebeck coefficient *S* of the SWCNT films was measured at near 300 K with an accuracy of ±10%. The film size was 25 mm × 20 mm and the visual film thickness was approximately 10 μm. One end of the film was connected to a heat sink and the other end was connected to a heater. Two 0.1-mm-diameter K-type thermocouples were pressed on the center of the thin films with a distance between them of 20 mm. The temperature difference between the thermocouples was varied from 1 to 4 K, while the Seebeck voltage was recorded at every 1 K (temperature reader: KEYENCE GR-3500 and digital multimeter: ADVANTEST R6561). The Seebeck coefficient was estimated from the V-K slope using the linear approximation. We measured the Seebeck coefficient four times per sample and calculated the average value. In order to measure the time-dependence of the Seebeck coefficient of the SWCNT films, the measurement was first performed at intervals of 1 day for a total of 7 days; thereafter, the measurement was performed at intervals of 7 days for a total of 63 days. The in-plane resistance of the SWCNT films was measured near 300 K using a four-point probe method (Napson, RT-70V) with an accuracy of ±10%.

### Characterizations

The surface and cross-section morphologies of the SWCNT films were observed using field emission scanning electron microscopy (FE-SEM; Hitachi S-4800). The chemical structures of the SWCNT films were characterized by Fourier transform infrared spectroscopy (FT-IR; JASCO FT/IR-4200), microscopic Raman spectroscopy (HORIBA XploRA), and X-ray photoelectron spectroscopy (XPS; ULVAC-PHI Quantum 2000).

## Supplementary information

Supplementary Information.

## Data Availability

The authors declare that most data supporting the findings of this study are available within the paper and its supplementary information files. The rest of the data are available from the corresponding author upon reasonable request.
