# Supplementary material for: Facile preparation of air-stable n-type thermoelectric single-wall carbon nanotube films with anionic surfactants
Source: Sci Rep. 2020 May 15;10:8104. doi: 10.1038/s41598-020-64959-5 (PMC7228955; doi:10.1038/s41598-020-64959-5)
Supplement: Supplementary file 1 — Supplementary Information. [file 41598_2020_64959_MOESM1_ESM.doc]

**Facile preparation of air-stable n-type thermoelectric single-wall carbon nanotube films with anionic surfactants**

Yuhei Seki, Kizashi Nagata and Masayuki Takashiri ^*^

*Department of Materials Science, Tokai University, Hiratsuka, Kanagawa 259-1292, Japan*

*E-mail: takashiri@tokai-u.jp

**Supplemental information**

Figure S1. Molecular structures of surfactants. (a) sodium dodecyl sulfate (SDS), (b) sodium dodecylbenzenesulfonate (SDBS), and (c) sodium cholate (SC).

Figure S2. Surface SEM image of the surfactant-free SWCNT film.

Figure S3. Cross-section SEM images of the SWCNT films with different surfactants and heat-treatment temperatures. (a) SDS-non heated, (b) SDS-150°C, (c) SDS-350°C, (d) SDBS-non heated, (e) SDBS-150°C, (f) SDBS-350°C, (g) SC-non heated, (h) SC-150°C, and (i) SC-350°C.

Figure S4. Wettability measurements of the different surfactant solutions.

Figure S5. Relationship between resistance of the SWCNT films with different surfactants and the heat-treatment temperature.

Figure S6. Chronological change in resistance of the SWCNT films with different surfactants. (a) SDBS and (b) SC.

Figure S7. Chronological change in power factor of the SWCNT films with SDBS as surfactant and heat treatment at 350°C. The power factor is estimated based on the visual film thickness determined by cross-section SEM images.

Figure S8. Schematic diagram of the dispersion liquid removal technique in (a) drop casting and (b) vacuum filtering.

Figure S9. Recovery of resistance of SWCNT films with SDBS as surfactant and heat treatment at 350°C.

Figure S10. Change in mass of the SWCNT films with SDBS as surfactant and heat treatment at 350°C.

Figure S11. Raman spectra of SWCNT films with SC and different heat-treatment temperatures. (a) Wavenumber ranging from 1000 to 2000 cm^−1^ and (b) wavenumber ranging from 1500 to 1700 cm^−1^ for precisely displaying the G-band spectra.


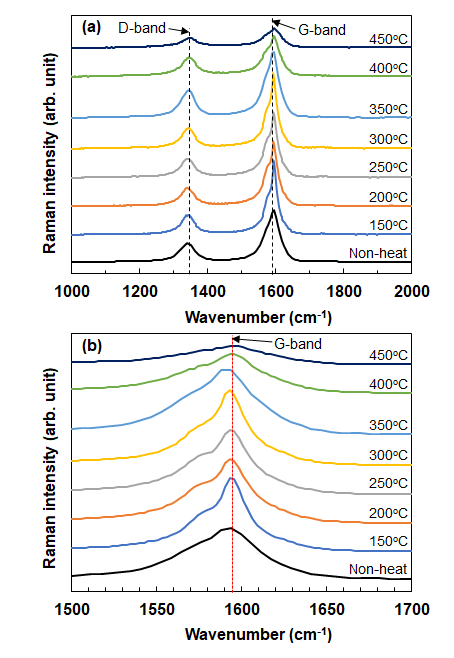


Figure S12. XPS spectrum of O1s in the surfactant-free SWCNT film with no heat treatment and that with heat treatment at 350°C.
